# Sequencing proteins with transverse ionic transport in nanochannels

**DOI:** 10.1038/srep25232

**Published:** 2016-05-03

**Authors:** Paul Boynton, Massimiliano Di Ventra

**Affiliations:** 1 University of California, San Diego, Department of Physics, La Jolla, CA, 92093-0319 USA

## Abstract

*De novo* protein sequencing is essential for understanding cellular processes that govern the function of living organisms and all sequence modifications that occur after a protein has been constructed from its corresponding DNA code. By obtaining the order of the amino acids that compose a given protein one can then determine both its secondary and tertiary structures through structure prediction, which is used to create models for protein aggregation diseases such as Alzheimer’s Disease. Here, we propose a new technique for *de novo* protein sequencing that involves translocating a polypeptide through a synthetic nanochannel and measuring the ionic current of each amino acid through an intersecting *perpendicular* nanochannel. We find that the distribution of ionic currents for each of the 20 proteinogenic amino acids encoded by eukaryotic genes is statistically distinct, showing this technique’s potential for *de novo* protein sequencing.

Living organisms depend on proteins to carry out the genetic code and perform many vital cellular tasks like metabolism^1^. To understand how a protein works one must understand its structure. Proteins are special because of how versatile they are in binding to other molecules, and the structure of these binding sites often indicate the precise use of a protein.

The first step in understanding protein structure is knowing the sequence of a protein, meaning the order of the amino acids that compose it. There are 20 amino acids that are used as building blocks by eukaryotic genes to make proteins, all of which have the same chain of atoms as a backbone. What distinguishes each amino acid is its side chain, which can span from a single hydrogen in the case of glycine (GLY) to containing an indole functional group in the case of tryptophan^1^. For a protein to function these amino acids fold up into secondary and tertiary structures that expose features like binding sites, which can be predicted based on the protein sequence. Ongoing research attempts to understand protein aggregation diseases such as Alzheimer’s Disease^2^ by performing simulations of structure formation, which would not be possible without the knowledge of the components of the peptides and proteins involved. In addition, protein sequences allow the synthesization of other proteins, which is necessary to compensate for diseases like Diabetes Type I in which the body does not produce the necessary peptide hormone insulin^3^,^4^.

The most common method for *de novo* protein or peptide sequencing (namely sequencing a protein for the first time) is mass spectrometry, a technique that involves fractionating the peptide into many smaller peptides and then obtaining the mass-to-charge ratio of each new peptide from the mass spectrometer. The problem with this technique is that fractionation is often carried out with gel electrophoresis, which is inherently slow^5^. In addition, fractionation must be repeated many times to obtain small enough peptides so that one can discern the composite amino acids from just the total mass-to-charge ratio^6^. Also, *de novo* sequencing is sometimes impossible with this technique since some amino acids have the same mass and charge (*e.g.*, leucine and isoleucine).

Edman degradation is another common method for *de novo* protein or peptide sequencing that utilizes repeated chemical washing and N-terminal cleaving to identify the sequence of amino acids one at a time^7^. However, Edman degradation suffers from the same issue of fractionation as mass spectrometry since devices can only reliably sequence peptides up to about 30 amino acids^8^. Nonetheless, the end result of identification via chromatography of each singled out chemically modified amino acid is reliable, albeit slow, but does require the use of many reagents.

The advent of nanopore DNA sequencing^9^,^10^ has brought several modern techniques to protein detection: longitudinal ionic transport^11^,^12^ and transverse electronic transport^13^. In the case of ionic transport through a single nanopore, detection of the protein folding state is achieved experimentally and modeled with exclusion volumes by^11^. Of course, protein sequencing with such a technique is a more difficult task and has not been achieved as of yet^12^. In fact, longitudinal ionic transport detects a current blockade which is the convolution of several blockade events from different amino acids^10^.

Transverse electronic transport, a technique in which amino acids are detected by a pair of electrodes transverse to peptide translocation, has been shown to be successful in identifying single amino acids and even in differentiating between tyrosine and phosphotyrosine^13^, a post-translational modification. However, only 12 of the 20 amino acids were able to be detected by this technique with two different electrode gap distances (0.55 nm and 0.7 nm) since the tunneling current is highly dependent on this gap distance and an amino acid’s ability to enter the gap. In other words, a single gap cannot be used for all amino acids.

This brings us to our proposed technique, sequencing proteins with *transverse ionic transport*. Like the two aforementioned techniques, this method is inspired by a DNA sequencing method^14^,^15^ and does not require reagents or fractionation since these devices do not place a limit on the length of the polypeptide^10^, meaning these nanopore techniques have the potential to be much faster. The structure of this proposed device is the same as in^14^,^15^, with a longitudinal nanochannel for polypeptide translocation and an intersecting transverse nanochannel for ionic transport driven by an electric field, 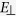, as in Fig. 1. However, the longitudinal nanochannel must be larger than in^14^ to accomodate the various sizes of the amino acids and instead of 4 DNA bases we need to distinguish 20 amino acids. Therefore, the molecular dynamics (MD) simulation method utilized in^14^ is time prohibitive for our purposes so we resort to a hard sphere model to account for the electrostatic properties of each amino acid, which requires only one MD run per amino acid to execute. Afterwards we use Monte Carlo sampling to calculate ionic current distributions based on external azimuthal rotations (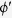) and dihedral angle (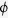 and *ψ* as in Fig. 2) distributions, or Ramachandran plots. We show that the distribution of ionic currents for each of the 20 proteinogenic amino acids encoded by eukaryotic genes is indeed statistically distinct, and propose a protocol for *de novo* protein sequencing based on this technique.

## Theoretical Approach

Let us then consider the configuration of crossed nanochannels we have in mind. Although not necessary for our conclusions, we assume for simplicity that the nanochannels have circular cross sections. We will discuss the suggested experimental preparation later in the manuscript.

**Figure 1:**
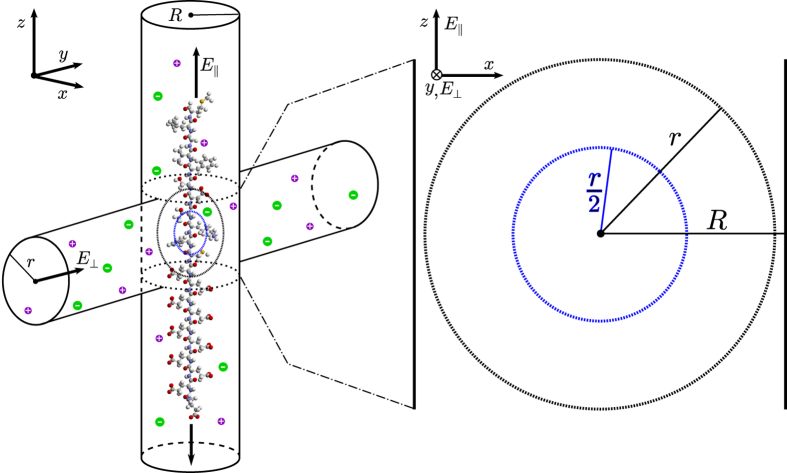
A schematic of the transverse ionic transport sequencing method. Two nanochannels intersect: the vertical or longitudinal channel along *z* with radius *R* and the horizontal or transverse channel along *y* with radius *r*. The polypeptide translocates along the longitudinal channel crossing the transverse channel that contains ions, purple K^+^ and green Cl^−^, that flow along the transverse channel due to an electric field, *E*_⊥_, in the +*y* direction. In this case the polypeptide consists of neurokinin A starting at the C-terminus at the top of the figure attached to one cysteine (CYS) followed by 10 glutamic acids (GLUs), a negatively charged amino acid, where the last GLU makes up the N-terminus (see later in text for more on this structure). This negatively charged polypeptide is driven towards *−z* by an electric field, *E*_∥_, in the +*z* direction. The dotted lines represent the top and bottom extremities of the intersection of the transverse channel, which are expanded to the right along with the thick dashed lines representing the area-limiting cross section (outer black line) and the Monte Carlo radial limit (inner blue line) that lie in the *xz*-plane. For visibility purposes the polypeptide is enlarged by a factor of 3 in both of its dimensions from the actual scale that we used in simulations while the ion radius is enlarged by a factor of 1.5.

The polypeptide of interest unfolds inside a nanochannel pulled with a longitudinal force, while it blocks the ionic current flowing in a transverse channel, as schematically shown in Fig. 1. We take this longitudinal force to be much less than the transverse force that drives the ions through the transverse nanochannel so that the amino acid resides in the region of nanochannel intersection long enough to obtain the necessary measurements of ionic current for identification. As a result we can assume that the longitudinal ionic flow is negligible when compared to the transverse ionic flow.

It is well understood that the hydration layers surrounding each amino acid have different binding energies^16^,^17^, which certainly affect the ionic transport transverse to each amino acid. In addition, the amino acid may attract or repel ions due to its solvated charge or polarity state^18^,^19^. In order to understand the aqueous environment of each amino acid and determine its effect on the ionic transport, we run MD simulations for each amino acid. We consider the system at normal human body temperature, 310 K, and the solvated system is large enough to make quantum effects negligible. This allows us to use classical molecular dynamics and employ the highly-parallel NAMD2^20^ to run all of our simulations.

The MD setup starts with a single amino acid isolated from a straight (dihedral angles *ψ* = *ϕ* = 180°) peptide chain, as in Fig. 1 with proline (PRO) as an exception, which is positioned so that the *z*-axis is the longitudinal axis. The rest of the MD methods can be found in the Supplementary Information.

The water padding is large enough in this system to examine proximal radial distribution functions (pRDFs) from the amino acid’s surface for K^+^ and Cl^−^ up to the point where the concentrations level out to the bulk values. We use the radius from the surface of the amino acid because the features in the concentration will be more prominent as opposed to using the radius from the origin, since the amino acids have irregular shapes. Similarly calculated pRDFs on DNA have been shown to be fairly accurate for reconstructing the surrounding solute even when combining all surface atoms’ pRDFs into one^21^,^22^, as is done in our calculations.

To obtain the pRDFs, we count the number of ions (for K^+^ and Cl^−^) in 0.5 Å thick shells starting from the surface of each amino acid, which is defined by the intersection of the composing atoms’ van der Waals (vdW) spheres. We then calculate the volume of each shell by subtracting the inner volume of the intersecting spheres from the outer volume, using a grid approximation with 0.1 Å sides for each volume calculation. With the number of ions and the volume of the corresponding shell we calculate the local concentration of K^+^ and Cl^−^ as a function of *r*_>_, taken to be the perpendicular distance from the vdW surface to the radial midpoint of the shell, from the first shell at *r*_>_ = 0.25 Å to the last at *r*_>_ = 44.75 Å, which is below the 4.8 nm upper bound of water padding.

**Figure 2:**
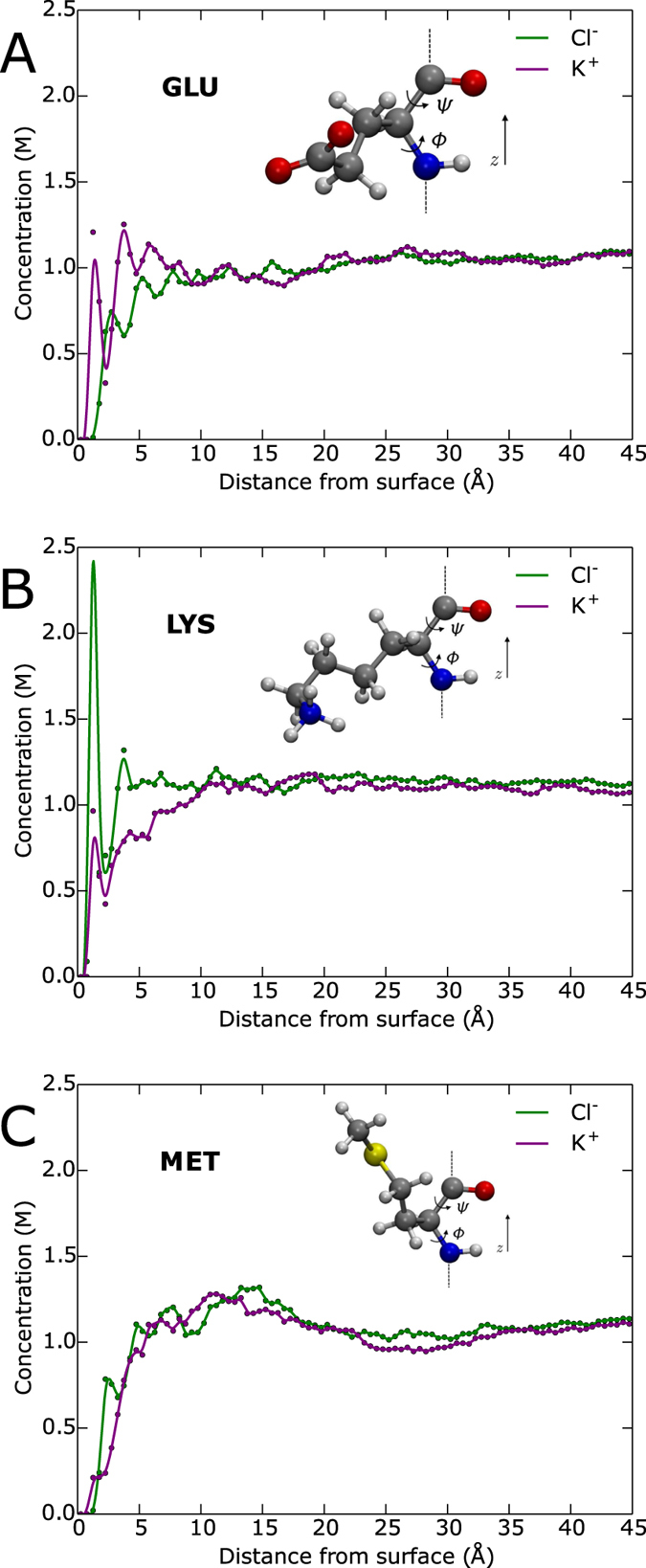
Plots of ionic concentration against distance from each amino acid’s vdW surface, *r*_>_, for amino acids GLU (plot A), LYS (plot B), and MET (plot C). K^+^ is represented by the purple line and Cl^−^ is represented by the green line. MD snapshots of each amino acid are included with labels for the direction of *z* and the dihedral angles *ϕ* and *ψ*.

As can be seen in Fig. 2, the concentrations reach a sufficiently steady bulk value at varying radii, with the maximum bulk *r*_>_ determined to be approximately 15 Å. Therefore we can focus on the part of the plots pertaining to *r*_>_ ≤15 Å to determine the solvation properties of each amino acid. As an example of our numerical procedure, we have chosen to feature the amino acid GLU in Fig. 2A, which has a negatively charged side chain at physiological pH (7.4), lysine (LYS) in Fig. 2B, which has a positively charged side chain at the same pH, and methionine (MET) in Fig. 2C, whose side chain is hydrophobic at this pH. These three amino acids are of similar size, which allows us to better compare the effects of charge states on transverse ionic current. We can immediately notice that the part of the pRDFs that we care about is quite different for each featured amino acid. GLU in Fig. 2A has a higher concentration of K^+^ due to its negativity while LYS in Fig. 2B has a higher concentration of Cl^−^ due to its positivity. Then there is the hydrophobic MET in Fig. 2C, which appropriately repels both K^+^ and Cl^−^ without much preference.

In the setting of an external electric field driving transverse ionic flow around an amino acid within a peptide, the potential barrier that ions must overcome in transport is influenced mostly by the electric potential in the neighborhood of the area-limiting cross section perpendicular to the ionic flow, imaged in Fig. 1 as the black thick-dashed line. This is partly due to the short interference time between the flowing ions and the circumvented amino acid. In our theoretical approach, we treat the equilibrium ionic concentrations as indicators of this electric potential to develop a hard sphere model with which we can calculate the distribution of ionic current for each amino acid. By calculating an effective radius, *r*_eff_, that is applied to every atom in the amino acid beyond its vdW radius, we can sample many amino acid orientations using a Monte Carlo approach to determine all of the ionic current distributions. We theorize that most of the variation in the transverse ionic transport will come from the exclusionary effects of the amino acid with respect to the direction of ionic flow, meaning that a large pool of orientations must be sampled to obtain an accurate view of these distributions.

Notice that by focusing on the area-limiting cross section perpendicular to the direction of ionic flow, 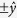, our theoretical approach is robust to the peptide chain’s translation along *y*, given that the two intersecting nanochannels have comparable radii. This is not necessarily the case for translation along *x*, yet we must take each amino acid to be centered in the nanochannel as in our MD preparation to satisfy the symmetry requirements of our hard sphere model. However, both the transverse and longitudinal external forces act to significantly reduce the variance in *x* while the transverse field additionally acts to center the peptide chain in *x*, which is further explained later in the manuscript.

In order to obtain the effective radius for each amino acid, we start with the definition of the average transverse ionic current of an ionic species *i*, K^+^ or Cl^−^ in our case, with valency 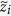 flowing around an amino acid.


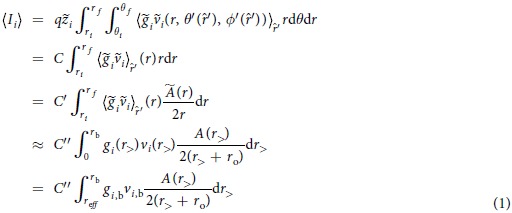


Here, the identifying transverse ionic current, *I*_*i*_, through the aforementioned area-limiting cross section perpendicular to the ionic flow, is averaged over all rotational orientations equally with 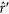 representing the unit 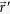 vector of the amino acid while *q* is the electron charge and *C*, *C*′, and *C*′′ are constants. 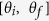 is the window of *θ* where the amino acid under study has non-negligible influence compared to neighboring amino acids. *r*_*i*_ is the radius where 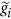, the local number density as a function of spherical coordinates, is first nonzero. *r*_*f*_ is the radius where the influence of the amino acid is no longer felt in the concentration and thus we need not continue the integral for the purpose of the effective radius, *r*_eff_, calculation. 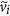 is the transverse velocity through the cross section as a function of standard spherical coordinates while 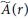 is the surface area of the sphere of radius *r*. In addition, *r*_>_ is the perpendicular distance from the vdW surface to the radial midpoint of a shell of thickness d*r*_>_ and surface area *A*, *r*_o_ is the average radius from the origin to the vdW surface, *r*_b_ is a value of *r*_>_ where the pRDF, *g*_*i*_ (plotted in Fig. 2), has become sufficiently steady around the bulk density, *g*_*i*,b_, so as to represent a shell in the bulk, *v*_*i*_ is the flow velocity of the ion species *i* as a function of *r*_>_, while *v*_*i*,b_ is the maximum of *v*_*i*_, which occurs in the bulk by construction.

The first approximation that we make is that all of the rotational orientations are uniformly likely, when in reality *θ*′ is fairly constant due to the stiffness of the peptide bond and given how small the diameter of the pore is in comparison to the length. However, when we average over 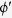 we fully explore the number density around the shell, so averaging over 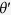 does not introduce any new data but adds more weight to the side chain as opposed to the ends of the backbone. This counteracts the simplification we make in our MD runs where we use isolated amino acids and include the number density at the ends of the backbone, which would normally be expelled by the nearest neighbor amino acids. Also, the internal dihedrals are assumed fixed since they do not fluctuate much under the imposed longitudinal electric field (see their implementation in the current distribution calculations). Lastly, when we change variables from *r* to *r*_>_ we have to approximate *r* as *r*_>_ +*r*_o_, which is a minor approximation when considering that all of the other functions in the integral have well-defined transformations. We can now use the following simplified equation to calculate the effective radius for our hard sphere model for every amino acid and ion species combination:


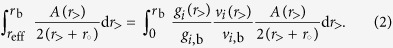


However, this equation requires the ratio of the transverse flow velocity compared to the bulk, and due to the small length scales we can use the Stokes equation, similar to^23^. The details of this calculation can be found in the Supplementary Information. From these calculations we find that *r*_b_ = (R−*r*_o_)/2 and then from our pRDF plots (see Fig. 2) we learn that the bulk concentrations start at approximately *r*_b_ ≥ 15 Å. Therefore for our model to work we have to take *R* ≥ 30 + max{*r*_o_} = 34.16 Å, where the max is over all amino acids, and then in the interest of minimizing the bulk ionic current we choose *R* = 35 Å. We also set the transverse nanochannel radius to the same value for simplicity.

**Figure 3:**
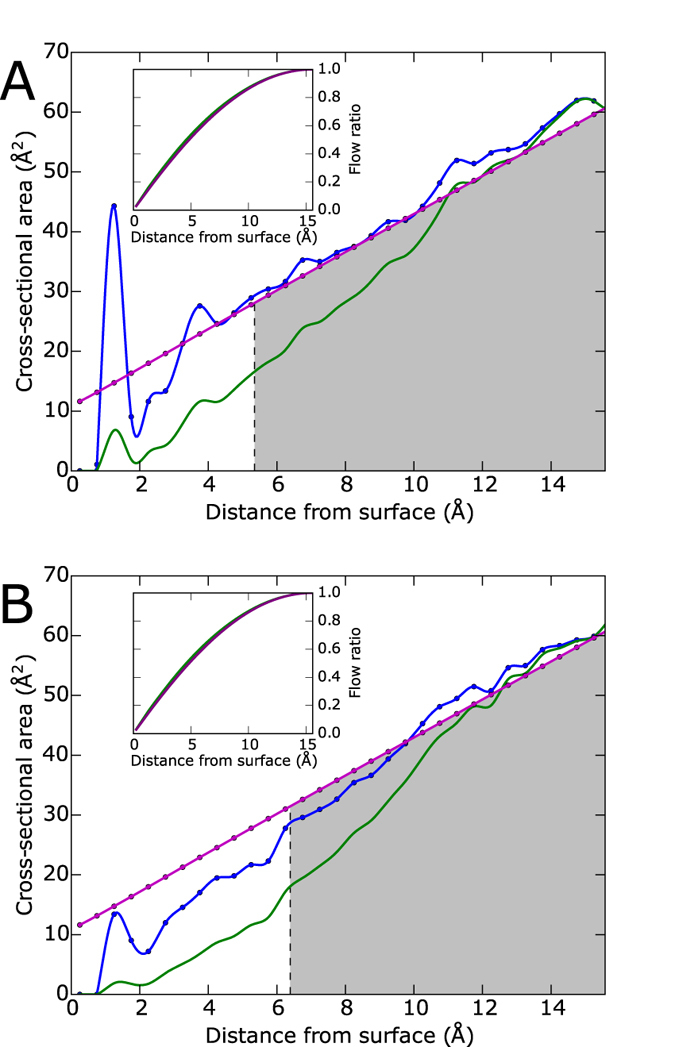
The top graph (plot A) represents area plots of Cl^−^ around LYS while the bottom graph (plot B) shows area plots of K^+^ around LYS. The straight magenta line is the average cross-sectional area that the shell of thickness Δ*r*_>_ = 0.5 Å at *r*_>_, the distance from the vdW surface of LYS, occupies in the plane *y* = 0. The blue line represents the average cross-sectional area that the ionic solution, with the number of ions from the shell of thickness Δ*r*_>_ = 0.5 Å at *r*_>_, would occupy in the plane *y* = 0 if those ions were reorganized to have bulk concentration, *g*_*i*,b_. The smooth green curve is the blue curve modulated by the ratio of the velocity with its maximum, *v*_*i*_/*v*_*i*,b_, which is plotted in the inset of each graph. The area under the smooth green curve is equal to the shaded gray area under the straight magenta curve while the dashed vertical line marks the effective radius for ion species *i* specifically for LYS.

The insets of Fig. 3 show the results of our calculations for *v*_*i*_/*v*_*i*,b_; Fig. 3A represents Cl^−^ around LYS while Fig. 3B shows K^+^ around LYS. The other amino acids have similar parabolic forms for *v*_*i*_/*v*_*i*,b_, but differing *r*_b_ because of differing *r*_o_. With *v*_*i*_/*v*_*i*,b_ calculated for every amino acid we can return to Eq. (2) to calculate our effective radii for our hard sphere model. This calculation is shown graphically in Fig. 3, where the straight magenta line is the argument (including d*r*_>_ as Δ*r*_>_ = 0.5 Å) of the left-hand side of Eq. (2), which is the average cross-sectional area that the shell of thickness Δ*r*_>_ at *r*_>_ occupies in the plane of interest (*y* = 0). The blue line represents the argument of the right hand side of Eq. (2), again including d*r*_>_ as Δ*r*_>_ = 0.5 Å without the modulation of the velocity ratio, leaving the average cross-sectional area that the ionic solution, with the number of ions from the shell of thickness Δ*r*_>_ at *r*_>_, would occupy in the plane of interest (*y* = 0) if those ions were reorganized to have concentration *g*_*i*,b_. Finally the smooth green curve is the blue curve modulated by *v*_*i*_/*v*_*i*,b_. The area under the smooth green curve is equal to the shaded gray area under the straight magenta curve, with the dashed vertical line marking not only where the shaded gray area ends on the left but also the effective radius for ion species *i* for the given amino acid. Because of the influence of the velocity, the fluctuations in concentration farther from the amino acid have more effect than closely bound spikes. For example, the Cl^−^ ion atmosphere located 1 Å from the surface of LYS has less effect on the effective radius compared to the next spike in concentration further out from the amino acid, as seen in the green curve. The fact that LYS is positively charged still shows in the effective radii though, with the attractive Cl^−^ ions having a 5.38 Å addition to the vdW surface compared to 6.43 Å for the repulsive K^+^ ions. The rest of the effective radii can be found in Supplementary Table S1.

This brings us to our Monte Carlo calculation of the transverse ionic current around each amino acid. Now that we have *r*_eff_ for each ion species that we add to the vdW radius of every atom in our amino acid, we can compare the available cross-sectional area through the *y* = 0 plane and apply the same bulk concentration and estimated bulk velocity, *g*_b_ = 1 M and *v*_b_, to all amino acids to obtain the ionic current values. We do not need to evaluate the available area in the entire cross section though since we only need to calculate up to the largest radius determined by *r*_eff_ for all amino acids. Therefore we use a radius of *R*/2 from the origin (see Fig. 1 where we are now limited to *r* = R) as the circular boundary for all of the amino acids since this circle encloses all of the extended amino acid surfaces in any applicable rotational configuration while also being enclosed by the bulk boundary defined by *r*_>_ = *r*_b_ where the velocity begins to decline from *v*_b_. We also approximate 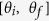 as 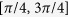 by comparing the backbone ends’ vdW radius to half of the distance in *z* between amino acids (half of ideally ~3.8 Å^24^). In this manner we can ignore portions of the cross section that would clearly be dominated by neighboring amino acids for the purposes of understanding each amino acid’s transverse ionic transport signature.

As previously mentioned, the current becomes sensitive to rotational conformations and dihedral angles in this portion of the calculation. Therefore, instead of assuming uniformity in 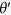 and straight dihedral angles like we did for the effective radius, we fix 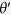 to 0 due to the rigidity of the peptide bond and we use Ramachandran plots^25^,^26^ to sample realistic values for 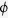 and *ψ*, dihedral angles as depicted in Fig. 2, according to a pulling force of 250 pN. This pulling force acts to straighten the polypeptide, which limits the available dihedral angle phase space and consequently reduces the variation in the *x* coordinate. Nevertheless, *ϕ* and *ψ* encompass the internal degrees of freedom for a chain of amino acids^26^,^27^. That leaves the azimuthal angle, 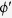, which we leave as uniformly distributed since as a whole the peptide does not have an azimuthal preference, except if the peptide is very short, in which case the transverse electric field that is only applied to a few amino acids can affect the entire chain.

We then apply Monte Carlo to a lone amino acid, the details of which can be found in the Supplementary Information. The reason we use a lone amino acid, the same one from our MD simulations, for calculating the ionic current distributions is that the first step to understanding the viability of this technique is distinguishing each amino acid separately via transverse ionic current. Since most of the exclusion due to the amino acid comes from the region of small *z*, where the uniqueness of the amino acid is demonstrated, the exclusion from one amino acid in a chain can be derived from our single amino acid distributions. As a result we do not treat the effect of neighboring PRO, which alters the dihedral angles so as to straighten the polypeptide chain. However, changing an amino acid’s dihedrals slightly does not change the ionic current distributions much since most of the variation in the current comes from azimuthal rotation of the amino acid.

Lastly, we must calculate the bulk velocity, *v*_b_, that we will use in the simple equation for the transverse ionic current, 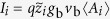 and 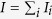, where 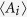 is the average area outside of the effective surface from Monte Carlo. This calculation can be found in the Supplementary Information, resulting in *v*_b_ = 77.23 m/s.

## Results and Discussion

**Figure 4:**
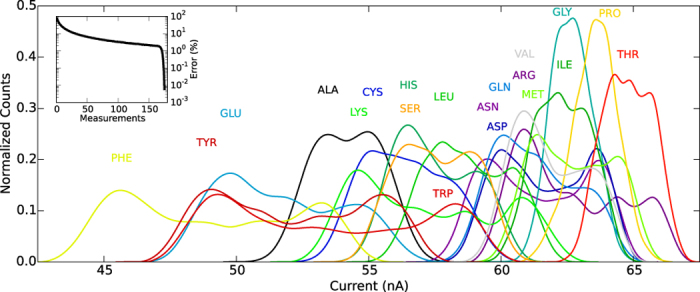
The transverse ionic current distributions for all 20 proteinogenic amino acids encoded by eukaryotic genes (identified with their standard three-letter abbreviations). The distributions have been normalized to the current values in nA. The inset plots the average error percentage over all 20 amino acids of identifying an amino acid correctly using *M* current measurements from that amino acid where the error percentage is on a log scale.

With a set of ionic currents for each amino acid determined from Monte Carlo utilizing our hard sphere model, we histogram each set of currents and use cubic spline interpolation to arrive at Fig. 4. The ionic currents tend to form multimodal (most often bimodal) distributions that are best described as a mixture of several normal distributions. The first and last peaks of each distribution tend to be the highest due to the variation in 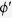. This is because the ionic current as a function of 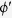 is roughly sinusoidal with a period of *π* and 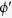 is uniformly distributed, which means the near minimum and near maximum values of the ionic current are chosen the most. Also due to the size of the nanochannels, the ionic current ranges in the tens of nA, which is well within the range of modern measurement devices that can resolve pA currents^15^,^28^. Beyond that, this ionic current only represents up to *R*/2 of the whole cross section. By using the parabolic 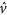 from the bulk region we calculate the contribution from the rest of the cross section, *r*_>_ >*r*_b_ but still within the *θ* limitations, as 69.86 nA after correcting the velocity for experiment. This value is comparable to the ionic current values from Fig. 4, meaning the distinctive component of the ionic current will not be dwarfed by the bulk in an experimental setting.

After a comparison of the ionic current distribution results with each amino acid’s vdW volume and isoelectric point (the pH at which an amino acid has neutral charge) we find that as expected an increase in vdW volume generally coincides with reduced ionic currents. For example, PRO and TRP both have close to neutral charge states so their size, PRO being small and TRP being large, completely determines their ionic current distributions. However, the isoelectric point obfuscates this expected trend in vdW volume by exhibiting a strong inverse relationship with the effective radius and therefore a direct relationship with ionic current. These relationships are best explained by the fact that a low isoelectric point, or a more negative charge state at neutral pH, attracts the positive side of the water molecule, which orients the hydrogens towards the amino acid and develops more tightly bound hydration layers as opposed to the reversed water orientation. As a result, the ions are supplanted further from the amino acid and subsequently there are fewer ions surrounding the amino acid, meaning the effective radius for both species of ions is increased while the allowed total transverse ionic current is decreased. GLU is a good example of an amino acid with a low isoelectric point, 3.15, that has much lower ionic current than amino acids of similar size (e.g. VAL). Whereas ARG, a positive amino acid with an isoelectric point of 10.76, has much larger ionic currents than the other large amino acids (e.g. TYR). The vdW volume does remain dominant in the standard deviation of the distributions, where the larger amino acids (ARG, PHE, TRP, TYR) find more variation in ionic current as the dihedrals or 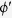 are altered.

These trends in the ionic current distributions do not hold true for all of the amino acids, however. ALA and SER both have unusually high effective radii for their size and isoelectric point while GLN, ILE, MET, THR, and ASN to a lesser extent have unusually low effective radii. SER, GLN, THR, and ASN encompass the proteinogenic amino acids with polar side chains so it’s not surprising that each of these amino acids displays deviation from the prior trends. Nevertheless, it is not obvious why ALA, ILE, and MET do not follow the aforementioned trends.

At a glance there is significant overlap between all of the distributions, yet the graph seems crowded mostly because of the sheer amount of plots to compare. We quantify the distinguishability of the ionic current distributions by calculating the error in selecting the correct amino acid, *X*, given *M* measurements from *X*. Based on the maximum likelihood decision rule^29^, the error is defined by


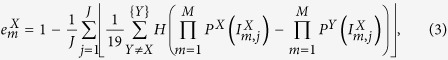


where *J* is the total number of realizations of the error calculation, {*Y*} is the set of all 20 amino acids, *H* is the Heaviside step function, and 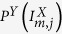 is the probability of 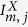, the *j* th realization of the *m*th ionic current measurement sampled from the current distribution for *X*, in *Y*′s ionic current distribution. Here, we assume that each measurement of ionic current is approximately independent. Next we average over *X* to obtain 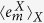 and then multiply by 100 to get the error percentage, which is plotted in the inset of Fig. 4. The error drops at a moderate rate with increasing *M*, but significantly drops off for *M* > 160 when the likelihood of at least one measurement giving zero probability to incorrect amino acids becomes very likely, making the product of those incorrect probabilities zero. For instance, at *M* = 175 the error percentage is practically 0%, and certainly less than 0.1%, a reasonable level of error. With a measurement frequency of 100 kHz^28^, and a best case scenario of 175 measurements per residue without any lapses in between, the sequencing rate becomes 571 residues per second.

## Sequencing Protocol

To build a nanofluidic device with intersecting channels as we suggest one may employ focused ion beam milling, as achieved in^15^ with two 10 nm diameter intersecting nanochannels. Our model requires two 7 nm diameter intersecting nanochannels, which is certainly achievable given that^30^ has shown non-intersecting sub-5 nm nanochannels from the focused ion beam milling technique. Although we have predicted that all 20 amino acids are statistically distinct within the framework of circular channels, other cross sections like rectangles or ellipses for the transverse channel allow fewer amino acids to blockade the ionic transport but still provide enough space for ions to flow past the translocating polypeptide. This results in improved residue selectivity and therefore decreased error as well as reduced post-processing time for deconvolution of the amino acid signals, which is necessary if more than one amino acid resides in the nanochannel intersection. Since the source of the distinguishability of the amino acids is their structural and electronic uniqueness we can assume that using a rectangular or elliptical transverse cross section with enough space along *x* for ionic flow would also result in 20 statistically distinct amino acids.

Once the sequencing device is built with transverse electrodes to control ionic flow, the protein or polypeptide of choice must be unfolded to translocate it through the longitudinal nanochannel. By using a high enough pulling force, around 250 pN^24^,^31^ that we also apply to our model, the polypeptide will unfold as well as translocate through the nanochannel. As opposed to chemical denaturing, force unfolding results in more confined and reliable Ramachandran plots^24^,^31^, which directly translates to more reliable ionic current distributions. After the polypeptide is unfolded the pulling force can be adjusted according to one’s ionic current measurement frequency and desired rate of error. For example, a desired 0.1% or less of error requires *M* = 175 and with a sequencing rate of 100 kHz as before, the maximum pulling speed would be 217 nm/s assuming an amino acid length of 3.8 Å. As a result, the maximum applicable pulling force would be ~180 pN^31^.

The next issue is then how this polypeptide is pulled through the nanochannel. As we have discussed, amino acids have varied charge states in solution. Therefore, to utilize an electric field for pulling (see Fig. 1) one has to attach charges to the polypeptide. These charges must be attached at the end of the chain so that one does not interfere with the ionic transport signatures of each amino acid. The best way to achieve this is by using a combination of solid phase peptide synthesis (SPPS), which excels at synthesizing smaller peptides^32^, and native chemical ligation (NCL)^33^ to attach a sequence of charged amino acids to the N-terminus of the polypeptide under study. We choose GLU as our charged amino acid because of how easily differentiable it is from the other amino acids (see Fig. 4) and how easy it is to produce. Using Fmoc, 9-fluorenylmethyloxycarbonyl or the chemical group that protects the N-terminus from reactions until desired, SPPS starting with N,N-bis(2-mercaptoethyl)-amide (BMEA)^34^ one creates a sequence of GLU with a length that will give the polypeptide chain plus GLU sequence a large enough charge to pull with an electric field. Fmoc SPPS is also used to attach a CYS residue to the N-terminus of the unknown polypeptide with a polyethylene glycol (PEG) support^35^. Then one uses NCL to take advantage of the transthioesterification reaction to form a native amide bond between the N-terminal CYS residue and the thioester precursor BMEA^34^.

An additional consequence of the attachment of charged residues onto the leading terminal of the polypeptide is that the transverse electric field acts to drive the polypeptide towards the boundary of the nanochannel aligned with one of the transverse nanochannel entrances. Therefore the polypeptide is pushed to the center of *x* and the variation in *x* is significantly reduced to match our theoretical approach. Upon reaching the transverse nanochannel, these leading charged residues are also pushed to potentially enter this channel, which can be prevented by stopping or reversing the transverse field upon detection of a partial blockade of the ionic current.

Another option is to use optical tweezers^36^,^37^ to target a terminal amino acid to pull the whole polypeptide. This approach has been utilized for longitudinal nanopore DNA sequencing^38^,^39^, resulting in more control over translocation due to the high tunability of optical tweezers. Advances in optical tweezers further allow a single beam to trap multiple targets^40^, potentially with computer-generated holograms^41^, which would allow even more control over the entire polypeptide.

For the sake of generality we do not specify a nanochannel material and so we do not include any adhesion or surface charge effects in our theoretical approach that are a result of this choice. However, these effects have a beneficial influence on sequencing by slowing or even controlling the translocation of the polypeptide^42^. These effects can also be used to better control the already small variation in *x*.

## Summary

We have proposed a novel *de novo* protein sequencing method in which an unfolded protein confined to a nanochannel is probed by transverse ionic transport through an intersecting nanochannel. This method promises to offer improved discrimination between amino acids by utilizing the 3-dimensional structure and electronic properties of each amino acid, as compared to techniques like mass spectrometry that can only probe total mass and charge^6^. We developed a hard sphere model for transverse ionic transport that employs the average equilibrium ionic concentrations surrounding all 20 amino acids derived from MD and ionic flow ratios determined by the Stokes equation. With this hard sphere model we were able to calculate distributions of ionic current for each amino acid based on Monte Carlo sampling of internal and external rotational conformations. All 20 amino acids were found to be statistically distinct and a sequencing error rate per residue of less than 0.1% was obtained with *M* = 175 measurements per amino acid, implying a best case scenario of 571 residues per second with a measurement frequency of 100 kHz^28^.

This approach is certainly experimentally achievable since 10 nm diameter intersecting nanochannels have been demonstrated for the purpose of DNA sequencing^15^ and polypeptides can be pulled through the nanochannel with optical tweezers or by adding charged residues to the polypeptide terminus and employing an electric field. Protein sequencing is very important since DNA sequencing cannot predict post-translational modifications and the ability to identify the sequence of a protein leads to the ability to understand its structure, which is the key to understanding many crippling diseases like Alzheimer’s^2^. We therefore hope our work will motivate the experimental realization of the proposed protein sequencing protocol.

## Additional Information

**How to cite this article**: Boynton, P. and Ventra, M. D. Sequencing proteins with transverse ionic transport in nanochannels. *Sci. Rep.* **6**, 25232; doi: 10.1038/srep25232 (2016).

## Supplementary Material

Supplementary Information

## References

[b1] Nelson D. L., Lehninger A. L. & Cox M. M. Lehninger Principles of Biochemistry (Macmillan, 2008).

[b2] Kelley N. W., Vishal V., Krafft G. A. & Pande V. S. Simulating oligomerization at experimental concentrations and long timescales: A Markov state model approach. J. Chem. Phys. 129, 214707 (2008). 19063575 10.1063/1.3010881 PMC2674793

[b3] Katsoyannis P. G. & Tometsko A. Insulin synthesis by recombination of A and B chains: A highly efficient method. Proc. Natl. Acad. Sci. 55, 1554 (1966). 5227674 10.1073/pnas.55.6.1554 PMC224358

[b4] Johnson I. S. Human insulin from recombinant DNA technology. Science 219, 632–637 (1983). 6337396 10.1126/science.6337396

[b5] Schmitt-Kopplin P. & Frommberger M. Capillary electrophoresis–mass spectrometry: 15 years of developments and applications. Electrophoresis 24, 3837–3867 (2003). 14661221 10.1002/elps.200305659

[b6] Standing K. G. Peptide and protein *de novo* sequencing by mass spectrometry. Curr. Opin. Struct. Biol. 13, 595–601 (2003). 14568614 10.1016/j.sbi.2003.09.005

[b7] Edman P. Method for determination of the amino acid sequence in peptides. Acta Chem. Scand. 4, 283–293 (1950).

[b8] Laursen R. A. Solid-phase Edman degradation. Eur. J. Biochem. 20, 89–102 (1971). 5578618 10.1111/j.1432-1033.1971.tb01366.x

[b9] Zwolak M. & Di Ventra M. *Colloquium*: Physical approaches to DNA sequencing and detection. Rev. Mod. Phys. 80, 141–165 (2008).

[b10] Branton D. *et al.* The potential and challenges of nanopore sequencing. Nat. Biotechnol. 26, 1146–1153 (2008). 18846088 10.1038/nbt.1495 PMC2683588

[b11] Talaga D. S. & Li J. Single-molecule protein unfolding in solid state nanopores. J. Am. Chem. Soc. 131, 9287–9297 (2009). 19530678 10.1021/ja901088b PMC2717167

[b12] Movileanu L. Interrogating single proteins through nanopores: Challenges and opportunities. Trends Biotechnol. 27, 333–341 (2009). 19394097 10.1016/j.tibtech.2009.02.008

[b13] Ohshiro T. *et al.* Detection of post-translational modifications in single peptides using electron tunnelling currents. Nat. Nanotechnol. 9, 835–840 (2014). 25218325 10.1038/nnano.2014.193

[b14] Wilson J. & Di Ventra M. Single-base DNA discrimination via transverse ionic transport. Nanotechnology 24, 415101 (2013). 24061386 10.1088/0957-4484/24/41/415101 PMC4516905

[b15] Menard L. D., Mair C. E., Woodson M. E., Alarie J. P. & Ramsey J. M. A device for performing lateral conductance measurements on individual double-stranded DNA molecules. ACS Nano 6, 9087–9094 (2012). 22950784 10.1021/nn303322r PMC3482132

[b16] Wolfenden R., Andersson L., Cullis P. & Southgate C. Affinities of amino acid side chains for solvent water. Biochemistry 20, 849–855 (1981). 7213619 10.1021/bi00507a030

[b17] Chang J., Lenhoff A. M. & Sandler S. I. Solvation free energy of amino acids and side-chain analogues. J. Phys. Chem. B 111, 2098–2106 (2007). 17269814 10.1021/jp0620163

[b18] Kish M. M., Ohanessian G. & Wesdemiotis C. The Na+ affinities of *α*-amino acids: Side-chain substituent effects. Int. J. Mass Spectrom. 227, 509–524 (2003).

[b19] Rulšek L. & Havlas Z. Theoretical studies of metal ion selectivity. 1. DFT calculations of interaction energies of amino acid side chains with selected transition metal ions (Co2+, Ni2+, Cu2+, Zn2+, Cd2+, and Hg2+). J. Am. Chem. Soc. 122, 10428–10439 (2000).

[b20] Phillips J. C. *et al.* Scalable molecular dynamics with NAMD. J. Comput. Chem. 26, 1781–1802 (2005). 16222654 10.1002/jcc.20289 PMC2486339

[b21] Rudnicki W. R. & Pettitt B. M. Modeling the DNA-solvent interface. Biopolymers 41, 107–119 (1997). 8986123 10.1002/(SICI)1097-0282(199701)41:1<107::AID-BIP10>3.0.CO;2-L

[b22] Feig M. & Pettitt B. M. Sodium and chlorine ions as part of the DNA solvation shell. Biophys. J. 77, 1769–1781 (1999). 10512802 10.1016/S0006-3495(99)77023-2 PMC1300463

[b23] Qiao R. & Aluru N. Ion concentrations and velocity profiles in nanochannel electroosmotic flows. J. Chem. Phys. 118, 4692–4701 (2003).

[b24] Stirnemann G., Kang S.-g., Zhou R. & Berne B. J. How force unfolding differs from chemical denaturation. Proc. Natl. Acad. Sci. 111, 3413–3418 (2014). 24550471 10.1073/pnas.1400752111 PMC3948277

[b25] Stirnemann G., Giganti D., Fernandez J. M. & Berne B. Elasticity, structure, and relaxation of extended proteins under force. Proc. Natl. Acad. Sci. 110, 3847–3852 (2013). 23407163 10.1073/pnas.1300596110 PMC3593838

[b26] Ramachandran G. & Sasisekharan V. Conformation of polypeptides and proteins. Adv. Protein Chem. 23, 283 (1968). 4882249 10.1016/s0065-3233(08)60402-7

[b27] Richardson J. S. The anatomy and taxonomy of protein structure. Adv. Protein Chem. 34, 167–339 (1981). 7020376 10.1016/s0065-3233(08)60520-3

[b28] Gao R., Ying Y.-L., Yan B.-Y. & Long Y.-T. An integrated current measurement system for nanopore analysis. Chin. Sci. Bull. 59, 4968–4973 (2014).

[b29] Duda R. O., Hart P. E. & Stork D. G. Pattern Classification (John Wiley & Sons, 2012).

[b30] Menard L. D. & Ramsey J. M. Fabrication of sub-5 nm nanochannels in insulating substrates using focused ion beam milling. Nano Lett. 11, 512–517 (2010). 21171628 10.1021/nl103369g PMC3125600

[b31] Carrion-Vazquez M. *et al.* Mechanical and chemical unfolding of a single protein: A comparison. Proc. Natl. Acad. Sci. 96, 3694–3699 (1999). 10097099 10.1073/pnas.96.7.3694 PMC22356

[b32] Merrifield R. B. Solid phase peptide synthesis. I. The synthesis of a tetrapeptide. J. Am. Chem. Soc. 85, 2149–2154 (1963).

[b33] Dawson P. E., Muir T. W., Clark-Lewis I. & Kent S. Synthesis of proteins by native chemical ligation. Science 266, 776–779 (1994). 7973629 10.1126/science.7973629

[b34] Hou W., Zhang X., Li F. & Liu C.-F. Peptidyl N,N-bis(2-mercaptoethyl)-amides as thioester precursors for native chemical ligation. Org. Lett. 13, 386–389 (2010). 21175148 10.1021/ol102735k

[b35] Roberts M., Bentley M. & Harris J. Chemistry for peptide and protein PEGylation. Adv. Drug Deliv. Rev. 64, 116–127 (2012). 10.1016/s0169-409x(02)00022-4 12052709

[b36] Ashkin A., Dziedzic J., Bjorkholm J. & Chu S. Observation of a single-beam gradient force optical trap for dielectric particles. Opt. Lett. 11, 288–290 (1986). 19730608 10.1364/ol.11.000288

[b37] Grier D. G. A revolution in optical manipulation. Nature 424, 810–816 (2003). 12917694 10.1038/nature01935

[b38] Keyser U. F. *et al.* Direct force measurements on DNA in a solid-state nanopore. Nat. Phys. 2, 473–477 (2006).

[b39] Trepagnier E. H., Radenovic A., Sivak D., Geissler P. & Liphardt J. Controlling DNA capture and propagation through artificial nanopores. Nano Lett. 7, 2824–2830 (2007). 17705552 10.1021/nl0714334

[b40] Arai F., Yoshikawa K., Sakami T. & Fukuda T. Synchronized laser micromanipulation of multiple targets along each trajectory by single laser. Appl. Phys. Lett. 85, 4301–4303 (2004).

[b41] Grier D. G. & Roichman Y. Holographic optical trapping. Appl. Opt. 45, 880–887 (2006). 16512529 10.1364/ao.45.000880

[b42] Luan B., Stolovitzky G. & Martyna G. Slowing and controlling the translocation of DNA in a solid-state nanopore. Nanoscale 4, 1068–1077 (2012). 22081018 10.1039/c1nr11201e PMC3543692

