# Supplementary material for: Sequencing proteins with transverse ionic transport in nanochannels
Source: Sci Rep. 2016 May 3;6:25232. doi: 10.1038/srep25232 (PMC4853742; doi:10.1038/srep25232)
Supplement: Supplementary Information [file srep25232-s1.pdf]

Sequencing proteins with transverse ionic transport in nanochannels

Paul Boynton[1,*] **and Massimiliano Di Ventra**[1]

[1]University of California, San Diego, Department of Physics, La Jolla, CA, USA 92093-0319
[*]pboynton@physics.ucsd.edu

ABSTRACT

De novo protein sequencing is essential for understanding cellular processes that govern the function of living organisms and all sequence modifications that occur after a protein has been constructed from its corresponding DNA code. By obtaining the order of the amino acids that composes a given protein one can then determine both its secondary and tertiary structures through structure prediction, which is used to create models for protein aggregation diseases such as Alzheimer's Disease. Here, we propose a new technique for *de novo* protein sequencing that involves translocating a polypeptide through a synthetic nanochannel and measuring the ionic current of each amino acid through an intersecting *perpendicular* nanochannel. We find that the distribution of ionic currents for each of the 20 proteinogenic amino acids encoded by eukaryotic genes is statistically distinct, showing this technique's potential for *de novo* protein sequencing.

Supplementary Information

Amino Acid	r_{eff} for Cl^-	r_{eff} for K^+
ALA	8.28	7.68
ARG	3.50	4.49
ASN	5.23	5.55
ASP	6.15	4.69
CYS	7.15	6.85
GLN	4.85	5.28
GLU	8.03	7.44
GLY	6.18	5.75
HIS	6.30	6.08
ILE	5.25	4.78
LEU	5.97	5.74
LYS	5.38	6.43
MET	4.92	4.73
PHE	7.87	7.84
PRO	5.02	4.43
SER	6.85	6.99
THR	4.15	4.57
TRP	6.59	6.74
TYR	6.93	7.29
VAL	5.37	5.27

Table S1. Effective radii in Å

Molecular Dynamics The amino acid is centered along the z-axis according to the geometric center in z of its terminal N and C atoms, while the molecule is centered in the xy-plane according to the geometric center in x and y of its terminal N atom and a nearest neighboring amino acid's terminal N atom. To fix the rotation angle between amino acids, as a convention, the terminal N atoms always have $y = 0$, as is the case in Fig. 1.

Since PRO has more rigid dihedral angles, we need to center it with the help of two neighboring GLYs, which have flexible dihedral angles, on each side of a single PRO. The nearest neighbor GLYs are configured to have dihedral angles that compensate for those of PRO while the farthest neighbor GLYs are configured to be straight, so that PRO and the two straight GLYs are directed along the longitudinal axis while the two straight GLYs are aligned in the xy-plane. As a result, we can center and then isolate PRO by using the usual centering method on the geometric average of the two straight GLYs, staying consistent with the choice of angles for the rest of the amino acids.

Once the amino acid is isolated, we solvate the system into a right hexagonal prism with regular hexagonal xy-planes having a height of 11 nm and an apothem of 5.9 nm to be used in NAMD2 with periodic boundary conditions in all three dimensions of space. This configuration gives every atom from every amino acid at least 4.8 nm of water padding in the unit cell, or in other words at least 9.6 nm of water between any atom and the closest atom in any neighboring periodic image. We then passivate and ionize the system to about 1 M of KCl, a typical biological solute. The size of the ions will certainly change the average local concentrations near the amino acid, which may then affect the ionic transport. We utilize the CHARMM22 with CMAP force field[1,2] for all of the amino acid, TIP3P water, and ion interactions. Each amino acid was held fixed throughout the run so that it would not diffuse around and the surrounding solution could equilibrate and be analyzed consistently. After equilibrating at 0 K and progressively ramping up the temperature to 310 K, the system is allowed to evolve in an NPT ensemble first for 1 ns followed by an NVT ensemble for 5 ns, all with 1 fs time steps and 1 ps coordinate recordings. The temperature is held fixed using a Langevin thermostat with a damping coefficient of 5 ps^{-1}. The first ns of the NVT production run is discarded as transient, leaving 4 ns of run time, or 4000 coordinate snapshots, to analyze radial concentration profiles.

Velocity calculation Due to the small length scales of the intersecting portion of our nanochannel system, we can use the Stokes equation,

$$\frac{\mathrm{d}}{\mathrm{d}x}\left(\mu\frac{\mathrm{d}}{\mathrm{d}x}\hat{v}_i(x)\right)+q\tilde{z}_i\hat{g}_i(x)E_\perp=0, \tag{S1}$$

where μ is the dynamic viscosity of the fluid, $E_\perp$ is the external electric field applied in the y direction, $\hat{v}_i$ is the transverse velocity through the cross section, $\hat{g}_i$ is the local number density, and ion-ion interactions are ignored. Here the flow velocity is independent of y due to the fact that the transverse nanochannel length is much larger than the diameter of the longitudinal nanochannel, $2R$, and that $2R$ is comparable to the diameter of the transverse nanochannel (as depicted in Fig. 1). Independence from z is similarly due to the longitudinal nanochannel's length being much larger than $2R$ but we must also choose R to be large enough for ions to diffuse along z after they enter the longitudinal channel at $y = \pm R$. This will make any variation along z have negligible impact on the end result of an average v_i over all rotational conformations. In our case we simply use the dynamic viscosity of water ($\mu = 7.5\times10^{-4}\,\mathrm{Pa\cdot s}$), even though the viscosity of water with ions will vary slightly,[3] and a reasonable value of $E_\perp = 5\times10^{8}\,\mathrm{N/C}$ taken from.[3] However, we must transform this equation into one that depends on $r_>$ to obtain v_i. Since v_i and g_i are averages over all rotational orientations, the problem is condensed to the region $x > 0$ and $[\theta_i, \theta_f]$. With θ_i and θ_f close enough to $\pi/2$, we can approximate $r_>$ as $x - x_\circ$, where $x_\circ$ is some constant, since at least for the amino acid backbone the contour lines of $r_>$ resemble those of x. With these approximations we obtain

$$\frac{\mathrm{d}}{\mathrm{d}r_>}\left(\mu\frac{\mathrm{d}}{\mathrm{d}r_>}v_i(r_>)\right)+q\tilde{z}_i g_i(r_>)E_\perp=0. \tag{S2}$$

This will give us the rough form of $v_i/v_{i,\mathrm{b}}$ between the following boundary conditions:

$$\begin{aligned} &v_i(r_>=0)=0\\ &v_i(r_>=\bar{r}_>=\frac{R-r_\circ}{2})\geq v_i(r_>). \end{aligned} \tag{S3}$$

$\bar{r}_> = (R - r_\circ)/2$ is approximately halfway between the vdW surface and the longitudinal nanochannel surface and is also our upper bound on $r_>$ as the domain of v_i. To obtain v_i for the entire range necessary for Eq. (2), we require that $r_\mathrm{b} = \bar{r}_> = (R - r_\circ)/2$ since $\bar{r}_>$ must be in the bulk as well. From our pRDF plots (see Fig. 2) we learn that the bulk concentrations start at approximately $r_> = 15$ Å, meaning that $r_\mathrm{b} \geq 15$ Å. Therefore for our model to work we have to take $R \geq 30 + \max\{r_\circ\} = 34.16$ Å, where the max is over all amino acids, and then in the interest of minimizing the bulk ionic current we choose $R = 35$ Å.

Monte Carlo The Ramachandran plots that we use in our Monte Carlo calculations account for a 250 pN longitudinal force that is applied to the polypeptide chain (ubiquitin and polyglycine in[4]) to pull it through the nanochannel. The pulling force acts to limit the phase space available to the dihedral angle pair (ϕ, ψ), making the configurations that are close to straight ($\psi = \phi = 180°$) much more appealing.[4] PRO and GLY have significantly different plots from the rest of the amino acids due to how the side chain of PRO bonds with its own amine nitrogen, part of the amino acid backbone, leading to restricted dihedrals while GLY has a hydrogen instead of a side chain leading to more freedom in the dihedrals. This way, while the rest of the amino acids are described by the Ramachandran plot of ubiquitin, which contains all 20 of the proteinogenic amino acids encoded by eukaryotic genes and well represents 18 of them, we describe PRO with the (ϕ, ψ) plot from the isolated PRO values within ubiquitin and GLY with the Ramachandran plot of the polyglycine analog of ubiquitin.[4] With these Ramachandran plots we use Monte Carlo sampling to obtain (ϕ, ψ) pairs that we then implement on a lone amino acid, where the number of realizations is dependent on the size of the domain of the Ramachandran plot (1408 realizations for ubiquitin). We also rotate the amino acid in $\phi' \in [0, 2\pi)$ by all multiples of $\pi/12$. Then the amino acid is projected onto the $y = 0$ plane and using Monte Carlo (1000 realizations) we calculate the area outside of the effective surface, called A_i, yet within either $\pi/2$ sector of radius $R/2$ centered around $z = 0$, where the ion i will fit according to its vdW radius.

Maximum velocity Since v_b is the maximum velocity between the amino acid and the longitudinal nanochannel surface as aforementioned, we can use Eq. (S1) to obtain the max of $\hat{v}$, which is equivalent to v_b. In this case we focus on the velocity within the bulk region, namely from the midpoint between the amino acid and the channel surface, $x = x_\mathrm{mid} = (R + r_\circ)/2$, to the channel surface, $x = R$. We employ the following boundary conditions,

$$\begin{aligned} &\hat{v}(x = R) = 0, \\ &\hat{v}(x = x_\mathrm{mid} = \frac{R + r_\circ}{2}) \geq \hat{v}(x), \end{aligned} \tag{S4}$$

which are very similar to Eq. (S3). By assuming a constant bulk concentration, g_b, over this region we quickly come to a parabolic solution to Eq. (S1) as well as determining $v_\mathrm{b} = q\tilde{z}g_\mathrm{b}E_\perp(R - r_\circ)^2/4\mu$, where $\tilde{z}_i$ has been simplified to $\tilde{z}$ since both ion species have the same valency. Then we have $v_\mathrm{b} = 154.47$ m/s by choosing a reasonable $r_\circ = 4$ Å, a necessity in making v_b independent of the amino acid under study, which is more likely in experiment. In fact, the absolute value of the velocity determined from the Stokes equation is known to differ from experiment,[3] as opposed to the velocity ratio that we have utilized thus far. However, these differences appear to be systematic,[3] and can be solved by dividing v_b in half, resulting in the corrected $v_\mathrm{b} = 77.23$ m/s.

References

1. Brooks, B. R. *et al.* CHARMM: The biomolecular simulation program. *J. Comput. Chem.* **30**, 1545–1614 (2009).
2. MacKerell, A. D., Feig, M. & Brooks, C. L. Extending the treatment of backbone energetics in protein force fields: Limitations of gas-phase quantum mechanics in reproducing protein conformational distributions in molecular dynamics simulations. *J. Comput. Chem.* **25**, 1400–1415 (2004).
3. Qiao, R. & Aluru, N. Ion concentrations and velocity profiles in nanochannel electroosmotic flows. *J. Chem. Phys.* **118**, 4692–4701 (2003).
4. Stirnemann, G., Giganti, D., Fernandez, J. M. & Berne, B. Elasticity, structure, and relaxation of extended proteins under force. *Proc. Natl. Acad. Sci.* **110**, 3847–3852 (2013).
